# A fresh look at immunocytometry’s role in personalized rheumatology

**DOI:** 10.1093/rheumatology/keaf362

**Published:** 2025-07-08

**Authors:** Erica Valencic, Alberto Tommasini, Andrea Taddio

**Affiliations:** Department of Paediatrics, Institute for Maternal and Child Health IRCCS Burlo Garofolo, Trieste, Italy; Department of Medicine, Surgery and Health Sciences, University of Trieste, Trieste, Italy; Department of Paediatrics, Institute for Maternal and Child Health IRCCS Burlo Garofolo, Trieste, Italy; Department of Medicine, Surgery and Health Sciences, University of Trieste, Trieste, Italy; Department of Paediatrics, Institute for Maternal and Child Health IRCCS Burlo Garofolo, Trieste, Italy; Department of Medicine, Surgery and Health Sciences, University of Trieste, Trieste, Italy

**This editorial refers to the article ‘Targeting interferon responses in juvenile dermatomyositis: Siglec-1 as an in vitro biomarker for JAK inhibitor efficacy’ by Saskia R. Veldkamp *et al*., 2025;64:5132–41.**

In this issue of Rheumatology, Veldkamp *et al.* demonstrated how it is possible to induce the expression of Siglec-1 on peripheral blood monocytes isolated from healthy donors and stimulated *in vitro* with exogenous recombinant stimuli (IFN-α, IFN-β and Toll Like Receptor agonists) [1]. This expression, evaluated through flow cytometry, can be inhibited by pretreating cells with various JAK pathway inhibitors, with effects that depend both on the kind of stimulation and on drug specificity and dose. Notably, plasma or serum samples from patients with disorders marked by the overexpression of the type I IFN (IFN-I) pathway (dermatomyositis, juvenile Systemic Lupus Erythematosus and COVID-19) were able to induce Siglec-1 production, akin to recombinant stimuli. A strong correlation between IFN-β concentrations in these samples and Siglec-1 levels on monocytes underscored the direct involvement of IFN-I in the observed effect.

Previous research had already established the measure of Siglec-1 expression on blood monocytes as a reliable alternative to interferon score calculations for assessing IFN-I-mediated inflammation in rheumatological patients [2, 3]. The Veldkamp study advances this by showing that patients’ sera can also stimulate Siglec-1 expression in healthy donor cells. This finding is particularly impactful because serum is a more convenient sample for preservation and centralized laboratory analysis compared with live peripheral cells. In addition, this research also offers a powerful *in vitro* model for studying IFN-I-mediated diseases and the involvement of monocytes in the inflammatory response [4]. The study also evidenced how flow cytometry can be used as an analytical method to quantify the biological activity of patient sera, highlighting its potential as a clinically meaningful tool for diagnosis, stratification and personalized treatment of rheumatic diseases. Direct measurement of soluble IFN-I in peripheral sera for clinical purposes is often unreliable due to their paracrine release, leading to barely detectable bloodstream cytokine levels. Veldkamp *et al.* demonstrated that, despite these low concentrations, the biological activity of IFN-I can still be profoundly significant, making the assessment of their activity in sera by flow cytometry highly valuable.

Further research is needed to determine whether measuring interferon activity in serum is interchangeable with assessing Siglec-1 expression directly on patient monocytes. Indeed, differences between the two approaches can highly impact results. For instance, cytometric analysis of peripheral blood cells from patients might offer heightened sensitivity compared with analysis of sera, as cells exposed to interferons in inflamed tissues may retain Siglec-1 expression longer, even after soluble interferon has cleared. Furthermore, variations in donor monocyte response to stimulation, whether interindividual or intraindividual, may compromise the assay’s reproducibility in assessing the biological activity of patients’ sera. This issue may be addressed by using commercially available reporter cells, such as modified Human Embryonic Kidney cells, which have shown efficacy in consistently quantifying interferon activity and differentiating the roles of various interferon types in inflammation [5].

The use of peripheral blood cells to directly or indirectly profile systemic inflammation, while not entirely new, remains a largely undervalued tool in rheumatology. Recent studies highlighted the promise of monocyte phenotypic profiling by flow cytometry in enhancing the evaluation of inflammatory responses in immunological diseases. Changes in Siglec-1 expression on monocytes have, for example, been shown to indicate IFN-I-related inflammation even when conventional inflammatory markers are negative [3]. This underscores the efficacy of comprehensive cellular analysis in revealing subtle yet crucial inflammatory markers.

Flow cytometry is a well-established analytical technique that has been clinically utilized since the 1980s for diagnosing and monitoring haematological and immunological diseases. It offers rapid, versatile, quantitative, and relatively low-cost analysis of cellular characteristics. By using fluorochrome-conjugated monoclonal antibodies, flow cytometry precisely identifies specific cell populations, can determine their activation state, cytokine profiles, and other functional markers. While high-sensitivity cytometry techniques are revolutionizing research by enabling comprehensive immune phenotyping in large patient cohorts and integrating data with bioinformatics, their immediate impact on single-patient profiling in routine clinical practice may be limited [6]. In this context, simpler assays measuring individual markers via flow cytometry can have a greater and more direct impact. Therefore, studies focusing on identifying and characterizing peripheral cell markers using flow cytometry are invaluable for advancing clinical diagnostics and personalized medicine in inflammatory and rheumatological disorders.

Flow cytometry can have several other applications to the field of rheumatology. For example, it can allow predicting and managing non-response to rituximab using B cell biomarkers in systemic lupus erythematosus [7]. In patients with secondary Hemophagocytic LymphoHistiocytosis (sHLH), including Macrophage Activation Syndrome (MAS), flow cytometry can reveal an increased frequency of CD4dimCD8+ T cells, expressing high levels of activation markers CD38 and HLA-DR. These cells are involved in MAS/sHLH pathogenesis and can provide valuable hints for differential diagnosis [8].

Flow cytometry can also help identify general immune defects that can underpin rheumatological conditions, such as B cell abnormalities, T cell dysregulation and myeloid cell defects. Some disorders, like Cytotoxic T-Lymphocyte-Associated protein 4 (CTLA4) and LPS-Responsive Beige-like Anchor protein (LRBA) deficiency or Signal Transducer and Activator of Transcription 1 (STAT1) Gain-of-Function, which can present with rheumatological complaints, can dramatically benefit from targeted treatments like abatacept or JAK inhibitors. This precision in diagnosis allows rheumatologists to select treatments that directly address the identified immune defect, leading to more effective and personalized medicine.

For many years, the rheumatological field largely overlooked the importance of immune cell profiles, likely due to the historical conviction that the pathogenesis of rheumatological disorders primarily stemmed from pathological antibodies. Only later did scientific research demonstrate how specific immune dysfunctions could underlie the development of many rheumatic conditions, thereby opening new avenues for diagnosis and treatment. As Dr R. Bucala of Yale School of Medicine observed, ‘Immunology in particular is the underlying basis for the science of rheumatology’. Given this foundational understanding, it is not surprising that the flow cytometric study of immune cells can significantly aid in classifying and stratifying rheumatological conditions.

The intertwined history of immunology and rheumatology exemplifies the convergence of disciplines, technologies and perspectives. It serves as a potent reminder that medical progress often stems not from revolutionary breakthroughs, but from the gradual recognition that seemingly disparate phenomena share common underlying mechanisms. Moving forward, this integrated understanding promises to yield treatments as sophisticated as the diseases they aim to combat.

## Data Availability

Data are available upon reasonable request by any qualified researchers who engage in rigorous, independent scientific research, and will be provided following review and approval of a research proposal and Statistical Analysis Plan (SAP) and execution of a Data Sharing Agreement (DSA). All data relevant to the study are included in the article.
